# Discriminating between the maxillary tuberosity and the alveolar tuberosity– a critical pictorial review

**DOI:** 10.1007/s00276-025-03569-0

**Published:** 2025-01-22

**Authors:** Carol Antonio Dandoczi, Mugurel Constantin Rusu, Alexandru Nicolae Mureşan, Răzvan Costin Tudose

**Affiliations:** 1 Division of Anatomy, Department 1, Faculty of Dentistry, “Carol Davila” University of Medicine and Pharmacy, Bucharest, RO-020021 Romania; 2 Research Department, “Dr. Carol Davila” Central Military Emergency Hospital, Bucharest, RO-010825 Romania

**Keywords:** Maxillary bone, Dentistry, Posterior superior alveolar nerve, Maxillary sinus, Alveolar bone

## Abstract

**Purpose:**

The maxillary tuberosity, a critical anatomical landmark in dentistry and maxillofacial surgery, is burdened by terminological confusion. This inconsistency hampers clinical practice and communication across disciplines.

**Method:**

Different resources were used to argue for the necessity of standardising the terminology related to maxillary tuberosity to enhance diagnostic precision and ultimately improve patient outcomes.

**Results:**

Most clinical and surgical studies dealing with the distal alveolar bone of the maxilla erroneously indicate it as “maxillary tuberosity”. By recognising the diverse definitions of this structure, errors and misinterpretation of studies could be reduced, and interdisciplinary collaboration could be improved. The term “alveolar tuberosity” is recommended to refer specifically to the distal end of the alveolar process of the maxilla. Anatomically, the maxillary tuberosity belongs to the body of the maxilla and forms part of the posterior wall of the maxillary sinus; therefore, it should not be located in the alveolar process.

**Conclusion:**

Adhering to the Terminologia Anatomica will clarify the critical clinical and surgical landmarks and enhance communication in clinical and academic settings.

## Introduction

The maxillary tuberosity (MT) is often referenced in dental and surgical literature, yet its definition remains inconsistent. This confusion is more than just academic; it can have real consequences in clinical practice. Terms like “posterior maxillary process”, “alveolar ridge extension”, and “maxillary tuberosity” are sometimes used interchangeably, leading to a lack of clarity in diagnosis, treatment planning, and communication among dental and surgical professionals. This article argues that a more standardised approach to this terminology is urgently needed to prevent clinical errors and unify dental and anatomical education.

From primary restorative dentistry to advanced surgical interventions, the increasing complexity of dental and maxillofacial procedures makes standardised anatomical terminology more urgent than ever. This is a matter of professional efficiency and a fundamental aspect of patient safety. A clear understanding of anatomical structures (defined by the Terminologia Anatomica) and their spatial relationships is crucial in medicine and dentistry for accurate diagnosis and effective treatment planning [36]. The 2nd edition of the Terminologia Anatomica locates the MT (tuber maxillae, eminentia maxillae) on the infratemporal surface of maxilla [30].

### Original anatomical evidence that issued the current opinion

**Fig. 1:**
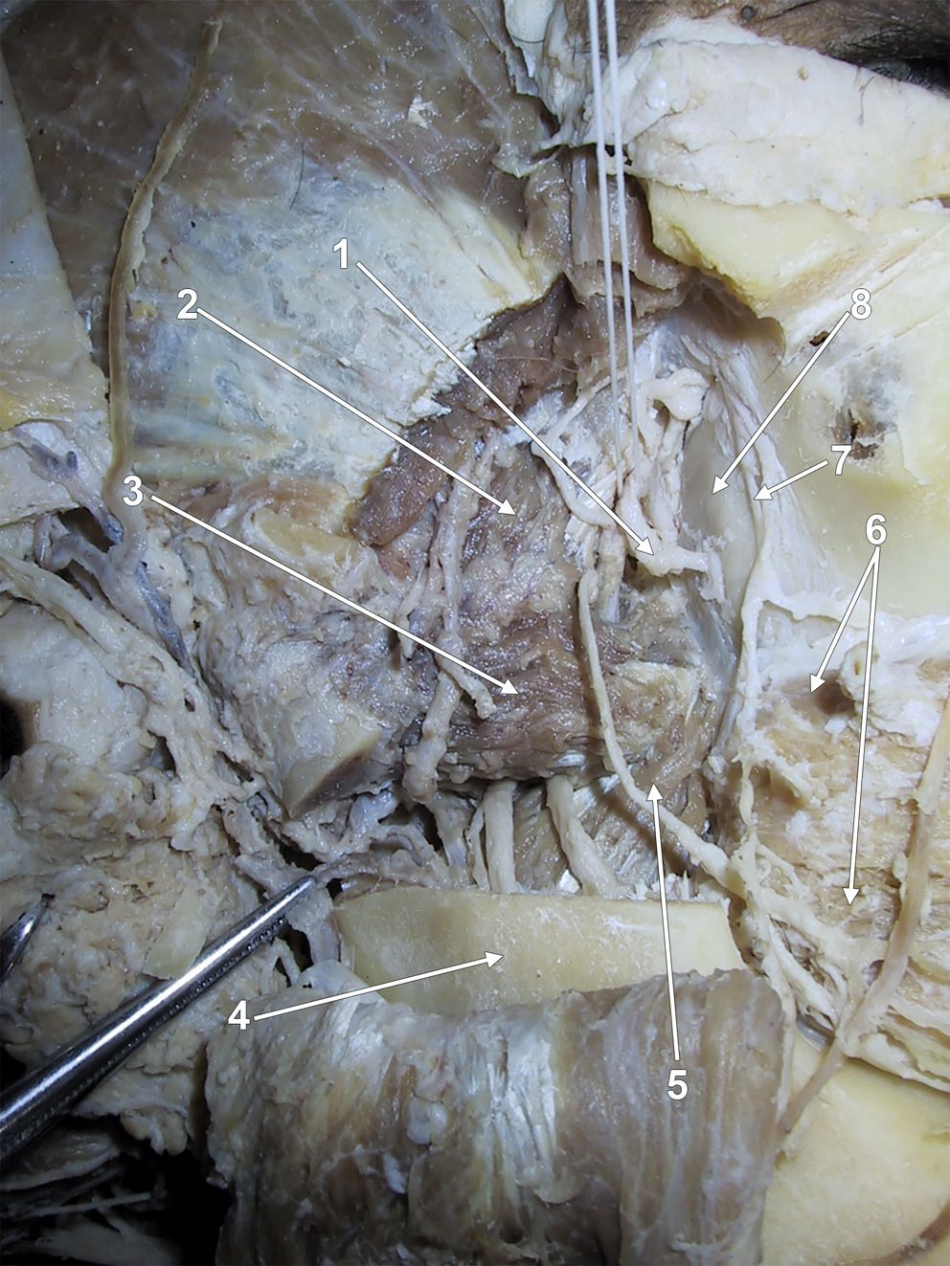
Dissection of the right infratemporal fossa. Lateral view. The temporal muscle tendon and coronoid process were removed. The medial pterygoid muscle’s superficial (accessory) head is attached to the maxillary bone’s alveolar process and the palatine bone’s pyramidal process. (1) posterior superior alveolar artery; (2) superior head of the lateral pterygoid muscle; (3) inferior head of the lateral pterygoid muscle; (4) mandibular ramus; (5) superficial (accessory) head of the medial pterygoid muscle; (6) buccinator muscle; (7) posterior superior alveolar nerve; (8) maxillary tuberosity

**Fig. 2:**
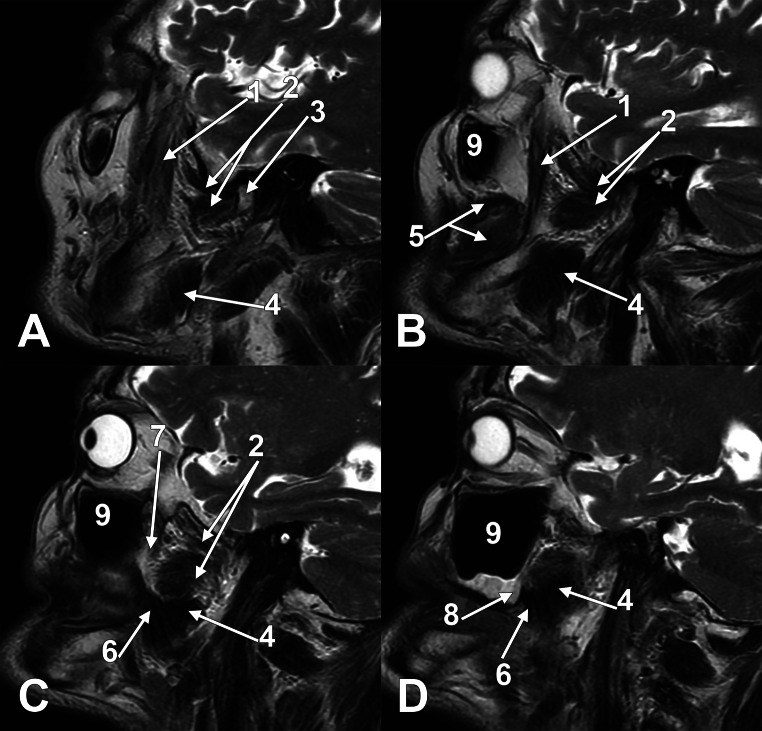
Lateral-to-medial series of sagittal MRI slices through the masticatory muscles. The medial pterygoid muscle’s superficial (accessory) head is attached to the distal maxillary alveolar bone. (1) temporal muscle (anterior bundle); (2) lateral pterygoid muscle; (3) head of the mandible; (4) deep (main) part of the medial pterygoid muscle; (5) buccinator muscle; (6) superficial (accessory) head of the medial pterygoid muscle; (7) posterior (infratemporal) face of the maxillary body; (8) distal end of the alveolar process of maxilla; (9) maxillary sinus

According to anatomical terminology, the maxillary bone comprises a body and processes. The processes are frontal, zygomatic, alveolar and palatine. The body has different surfaces: anterior, nasal, orbital, and infratemporal. The latter faces the infratemporal and pterygopalatine fossae and has a thin-walled MT on the posterior wall of the maxillary sinus [6]. We were intrigued when we checked the origin sites of the medial pterygoid muscle (MPM). Gray’s Anatomy details that the major component of the MPM „is the deep head that arises from the medial surface of the lateral pterygoid plate of the sphenoid bone and is therefore deep to the lower head of lateral pterygoid” and the small, superficial head of the MPM „arises from the maxillary tuberosity and the pyramidal process of the palatine bone, and therefore lies on the lower head of lateral pterygoid” [28]. However, when studying the figure related to this description, we observed that the bony attachment of the superficial or accessory fibres of the MPM does not raise superiorly to the buccinator muscle’s attachment on the alveolar process of the maxilla. Therefore, they do not ascend on the body of the maxillary bone to approach the pterygomaxillary fissure between the infratemporal and pterygopalatine fossae. We further checked this detail on original dissections (Fig. 1) and MRI successive sagittal slices (Fig. 2), and we demonstrated that the superficial (accessory) fibres of the MPM attach to the pyramidal process of the palatine bone (PP) and on the distal end of the alveolar process of the maxillary bone. We consider that the distal end of the maxillary alveolar bone could be referred to as the “alveolar tuberosity” (AT) to discriminate it from the MT on the body of the maxilla.

### The French anatomical description

**Fig. 3:**
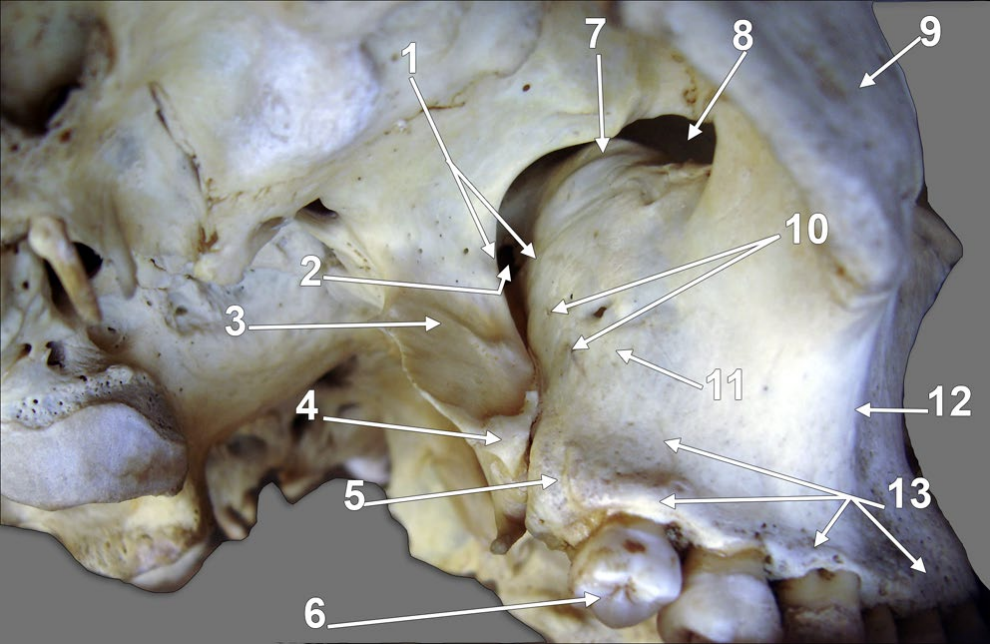
Dry skull. Postero-infero-lateral view of the right maxillary bone. (1) pterygomaxillary fissure; (2) pterygopalatine fossa; (3) lateral pterygoid plate; (4) pyramidal process of the palatine bone; (5) distal end of the alveolar process (alveolar tuberosity); (6) third maxillary molar; (7) sulcus of the maxillary nerve; (8) inferior orbital fissure; (9) zygomatic bone; (10) foramina of the posterior superior alveolar nerves; 11. maxillary tuberosity; 12. zygomaticoalveolar (jugal) crest; 13. alveolar process

As a sample for the French descriptions, we quote here Rouviére and Delmas who described that the posterior face of the maxillary body “*forme la paroi antérieure de la fosse ptérygomaxillaire et son arrière-fond. Elle est convexe dans sa partie interne; elle devient concave transversalement*,* en dehors*,* près de l’os malaire. La partie interne*,* convexe*,* saillante*,* est appelée tubérosité du maxillaire supérieur. On voit*,* dans sa partie moyenne*,* les orifices des canaux dentaires postérieurs*,* au nombre de deux à trois*,* dans lesquels s’engagent les vaisseaux et nerfs dentaires postérieurs. La partie la plus interne est rattachée par certains au bord postérieur de l’os. La face postérieure de la tubérosité est creusée*,* en haut*,* près du bord supérieur*,* d’une gouttière transversale dont la profondeur augmente de dedans en dehors jusqu’à l’extrémité postérieure de la gouttière sous-orbitaire. Cette gouttière répond au nerf maxillaire supérieur*“ [25]. The French description of the MT seems suitable and fitted to the anatomical observation. In Fig. 3, it can be observed that the MT is part of the body of the maxilla and contains alveolar foramina, whereas the AT is simply the distal end of the alveolar process. If the anatomical distinction between the body and the alveolar process of the maxilla is not ignored, the morphology of the maxilla is quite clear.

### The English basic description

**Fig. 4:**
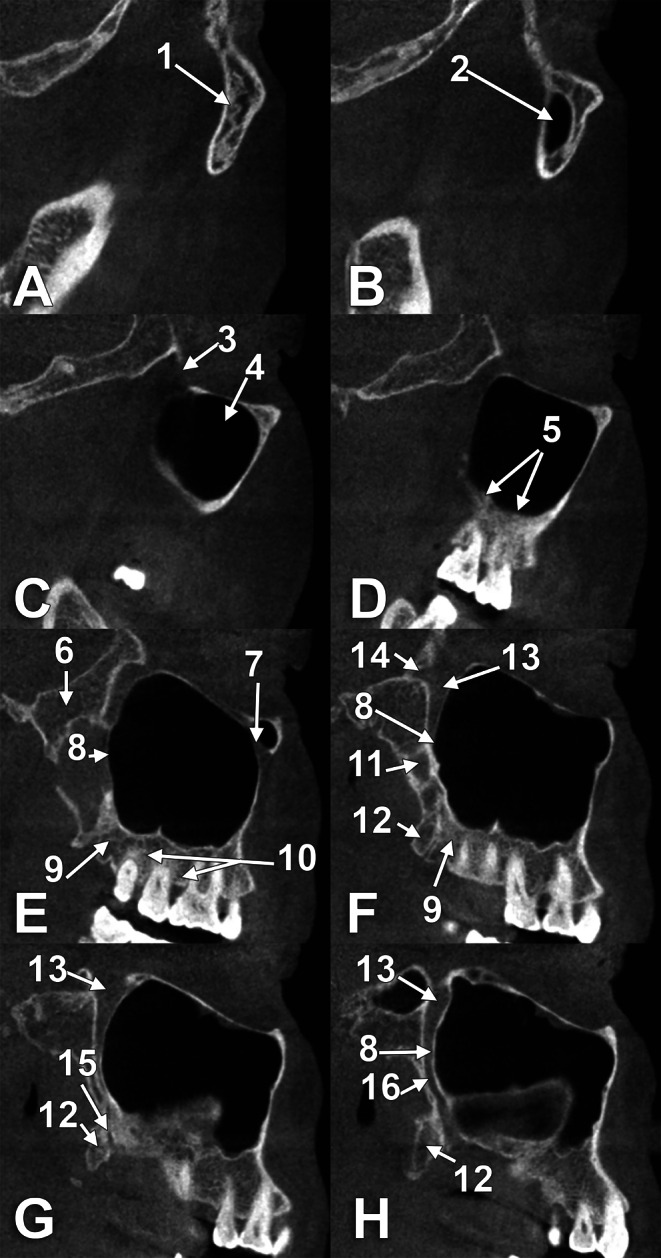
Lateral-to-medial series (**A**-**H**) of sagittal CBCT slices through a dentate right maxilla, viewed laterally. (1) zygomatic bone; (2) zygomatic recess of the maxillary sinus; (3) inferior orbital fissure; (4) maxillary sinus (antrum of Highmore); (5) antral floor; (6) non-lamellar root (base) of the pterygoid process; (7) infraorbital canal; (8) maxillary tuberosity; (9) distal end of the alveolar process (alveolar tuberosity); (10) alveolar process of the maxillary bone; 11. lateral pterygoid plate; 12. pyramidal process of the palatine bone; 13. pterygopalatine fossa; 14. foramen rotundum; 15. lesser palatine canal; 16. greater palatine canal

In Gray’s Anatomy, the general structure of the maxilla lists the body and processes [28]. However, when the infratemporal surface of the body of the maxilla is further described, it is mentioned that “posteroinferior is the MT, roughened superomedially where it articulates with the pyramidal process of the palatine bone” [28]. Further, it is described that „above the tuberosity, the smooth anterior boundary of the pterygopalatine fossa is grooved by the maxillary nerve as it passes laterally and slightly upwards into the infraorbital groove on the orbital surface”. When observing a dry skull (Fig. 3) and the sulcus of the maxillary nerve located just above the MT, it will be evident that the MT is on the body of the maxilla and does not articulate with the pyramidal process of the palatine bone. In line with the pyramidal process is the AT (Fig. 4).

### The alveolar tuberosity is made-up by alveolar bone

**Fig. 5:**
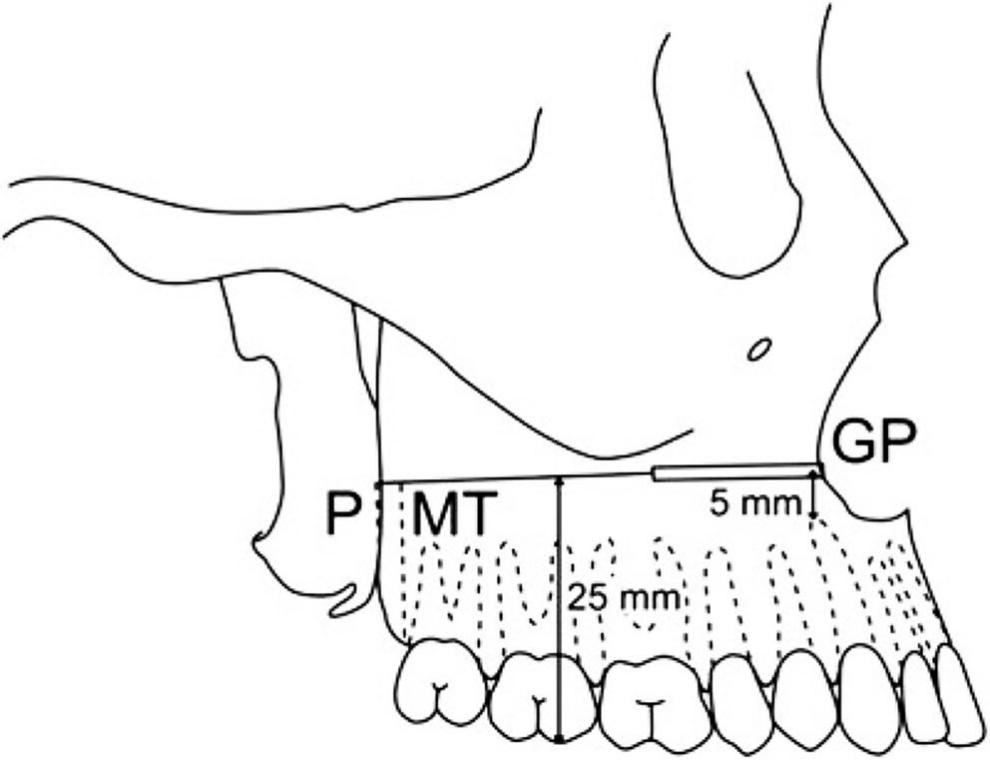
Image reused with the License 5,937,100,747,640/27 December 2024 provided by John Wiley and Sons and Copyright Clearance Center, from [3]. The original legend is “Fig. 1. Simulation of Le Fort I osteotomy. Gutta percha point (GP) adhered to the lateral maxillary osteotomy at Le Fort I level. MT, maxillary tuberosity cut; P, pterygomaxillary dysjunction”. The distal alveolar bone with molar roots is regarded there as maxillary tuberosity

**Fig. 6:**
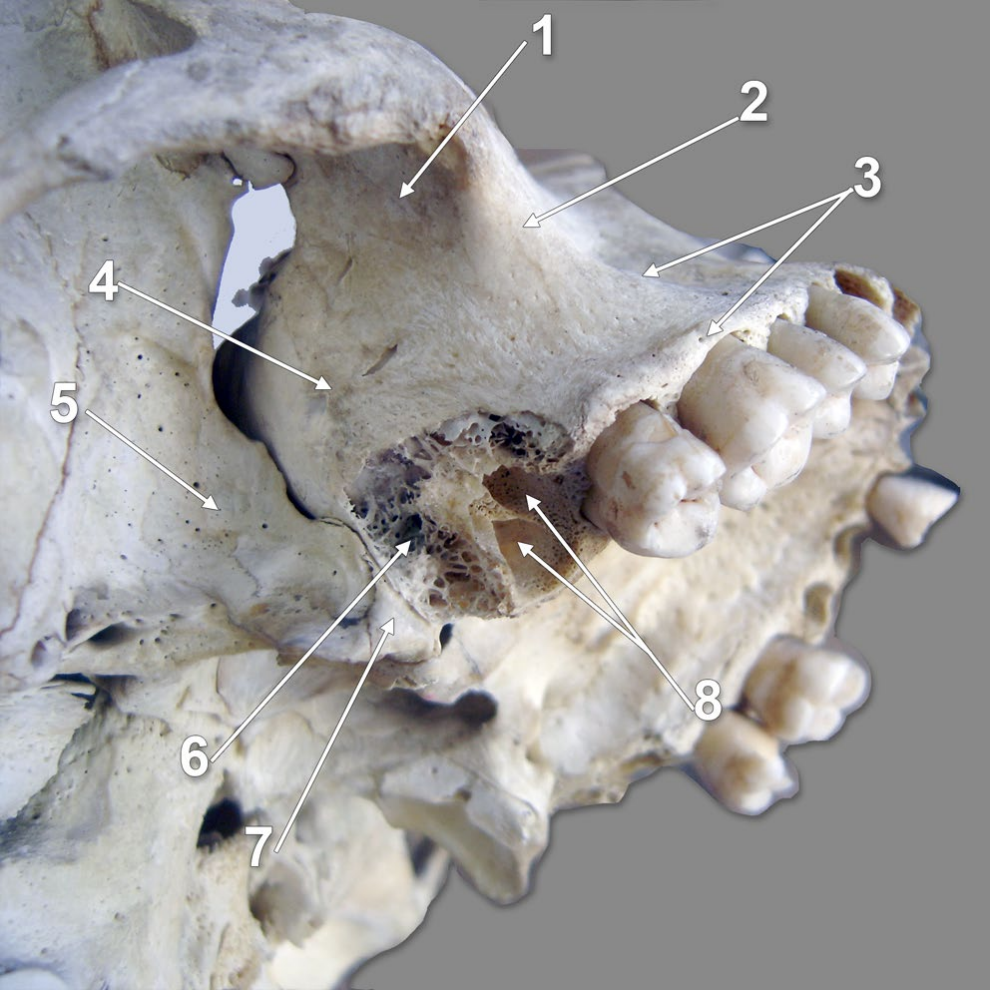
Dry skull. Postero-infero-lateral view of the right maxillary bone. (1) zygomatic process; (2) jugal or zygomaticoalveolar crest; (3) alveolar process; (4) maxillary tuberosity; (5) lateral pterygoid plate; (6) distal alveolar bone (cancellous bone of the alveolar tuberosity); (7) pyramidal process of the palatine bone; (8) radicular sockets of the third upper molar

For an anatomical study, Manzanera et al. (2018) used cone-beam computed tomography (CBCT) to examine the MT [22]. The authors defined there the MT as „the posterior extension of the maxillary bone, bounded mesially by the last erupted molar and maxillary sinus, and distally by the pterygopalatine fissure and pyramidal process of the palatine bone” by quoting an anterior study by Apinhasmit et al. (2005) [3]. Apinhasmit et al. (2005) did not use the term “pterygopalatine fissure”, which is erroneous, but they referred in their study to the “pterygopalatine fossa”, which is anatomically correct [3]. This last study was carefully reviewed, and Fig. 1 in that article (Fig. 5) clarified the authors’ interpretation of the MT. They regarded the AT we defined here as MT, which, in turn, they locate equally on the body and the alveolar process of the maxilla. Also, other authors perpetuated this inexact description. Manojna et al. (2023) introduced their recent study by discussing that orthodontic procedures, such as maxillary arch distalization, could only be performed when adequate space is available in the tuberosity area [21]. The authors affirmed that a thorough understanding of MT anatomy, dimensions, bone type, and surrounding structures is essential, especially when performing maxillary teeth distalization in orthodontic therapy [21]. However, they quote Vardimon et al. (2010) [35] when describing the MT as “a rounded projection of a compact bone that extends posteriorly from the alveolar crest, continuing the structure of the maxillary bone” and “is mesially bounded by the last erupted molar and maxillary sinus, while distally it is bounded by the pyramidal process of the palatine bone and pterygopalatine fissure” [21]. However, Vardimon et al. (2010) indicated that the distal limit of the MT is given by the pyramidal process of the palatine bone and the pterygomaxillary fissure [35] and not the pterygopalatine fissure, which is an erroneously used anatomical term by Manojna et al. (2023) [21]. When observing a dry skull (Fig. 6), one can notice that between the distally erupted molar and the pyramidal process of the palatine bone is nothing but the distal end of the alveolar process of the maxilla we termed AT; that alveolar bone is made up of trabecular bone covered by cortical bone. Moreover, if a specific area extends “posteriorly from the alveolar crest”, as Manojna et al. (2023) described, it could not be bounded mesially by the maxillary sinus.

**Fig. 7:**
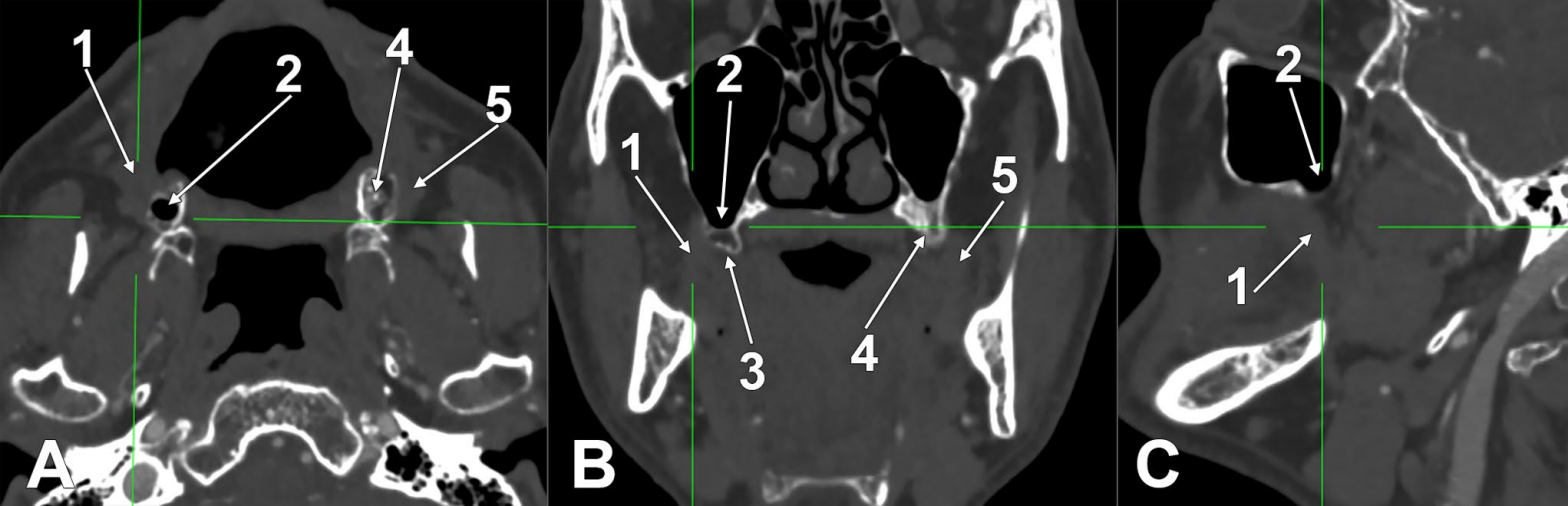
Residual alveolar tuberosity (AT) in corresponding CBCT slices. **A**. Axial slice, inferior view. **B**. Coronal slice, anterior view. **C**. Sagittal slice, medial view. (1) right buccinator muscle; (2) distal alveolar recess of the right antral floor, pneumatizes the right AT; (3) right AT; (4) left AT; (5) left buccinator muscle

Since the alveolar bone encases the roots of the teeth, it is logical to expect an increase in the volume of the distal end of the alveolar process of the maxillary bone—termed here the AT—during the development of the roots of the maxillary molars. Indeed, it was observed that the growth peak at this level occurs with the initiation of root development of the second upper molar and shortly before its eruption [35].

While describing a new surgical technique, the authors described how they harvested the residual bone in MT as a whole [2]. As the AT consists of alveolar bone that may resorb in edentulous cases, the distal end of the alveolar ridge in edentulous cases should be regarded as residual AT. In such cases (Fig. 7), a well-pneumatized residual AT will have an almost null height, and the buccinator muscle’s superior attachment will glide on the vestibular side of the antral floor. Therefore, the lack of documenting a residual AT before surgeries and approaching it represents a significant risk to establishing an oroantral communication.

### The nerves and arteries are related to the maxillary tuberosity

The maxillary nerve block has been described using two intraoral techniques, the greater palatine canal approach and the high tuberosity approach, which places the needle around the posterior maxilla until the needle enters the pterygopalatine fossa [7]. This locates the MT on the posterior antral wall. The posterior superior alveolar nerves have a descending course on the MT, not the AT (Fig. 1), as depicted in Malamed’s Handbook of Local Anesthesia [20]. However, the author used an image of a dry skull from McMinn’s Color Atlas of Human Anatomy [1], where the MT is indicated just as the “infratemporal (posterior) surface of maxilla”. At the same time, the distal alveolus wall of the third upper molar is marked as “tuberosity of maxilla” [20].

Thangavelu et al. (2012) indicated that posterior superior nerve anaesthesia requires navigating to the MT with a curved needle path [31]. Singla et al. (2015) demonstrated that the same results are obtained in the straight needle technique [27]. This shows that it is essential to deposit the anaesthetic substance in the posterior superior alveolar nerve area, thus at the alveolar foramina of the MT. Miscommunication about the exact location and structure of the MT could result in incorrect anaesthetic administration.

The lack of standardisation in the terminology surrounding MT has significant clinical implications. When performing posterior superior alveolar nerve blocks, precise identification of the MT is essential for avoiding nerve damage; however, when the tuberosity is misidentified– whether because of unclear anatomical references or misunderstood terminology– patients are at risk for nerve injury or insufficient anaesthesia.

**Fig. 8:**
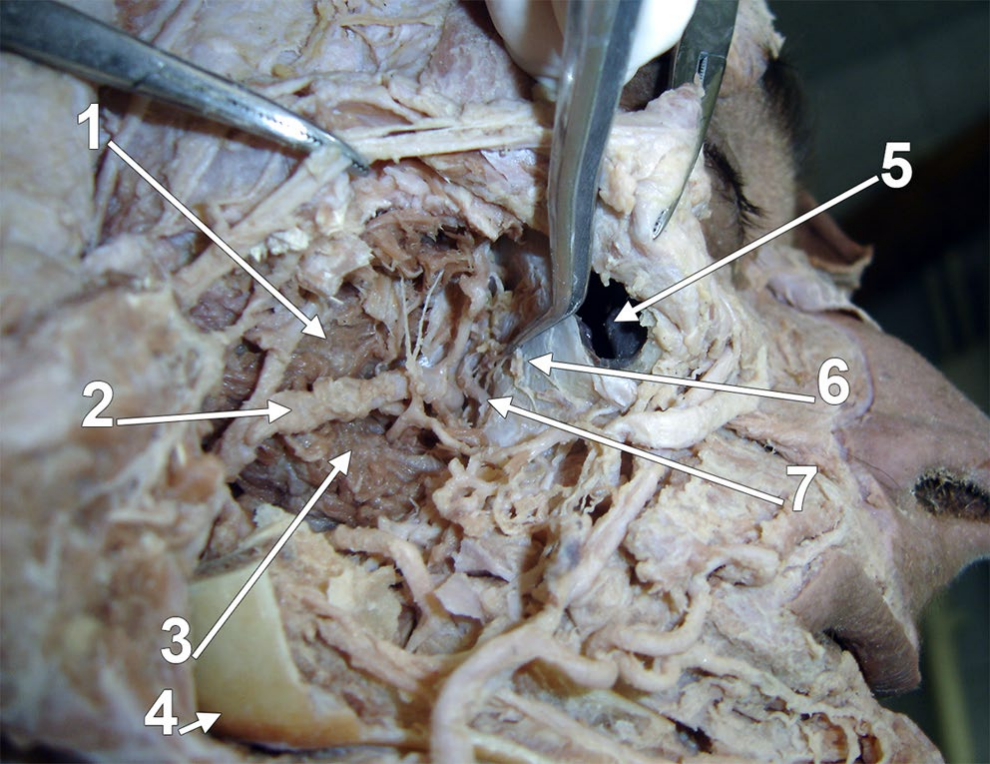
Dissection of the right infratemporal fossa. Lateral view. The maxillary artery is closely related to the maxillary tuberosity but not to the alveolar tuberosity. (1) superior head of the lateral pterygoid muscle; (2) maxillary artery; (3) inferior head of the lateral pterygoid muscle; (4) gonial angle; (5) maxillary sinus; (6) maxillary tuberosity; (7) posterior superior alveolar artery

When assessing the potential variations of the maxillary artery in relation to the MT, Uchida et al. (2022) observed the maxillary artery about the posterior antral wall rather than in association with the distal end of the alveolar process [33]. Attempting to identify the maxillary artery at the level of the distal alveolar arch of the maxilla would pose unnecessary risks for the surgeon. The maxillary artery reaches between the two heads of the lateral pterygoid muscle, superiorly to the maxillary attachment of the buccinator muscle, being thus related to the MT and not the AT (Fig. 8).

### The alveolar and maxillary tuberosities as related to the maxillary sinus

**Fig. 9:**
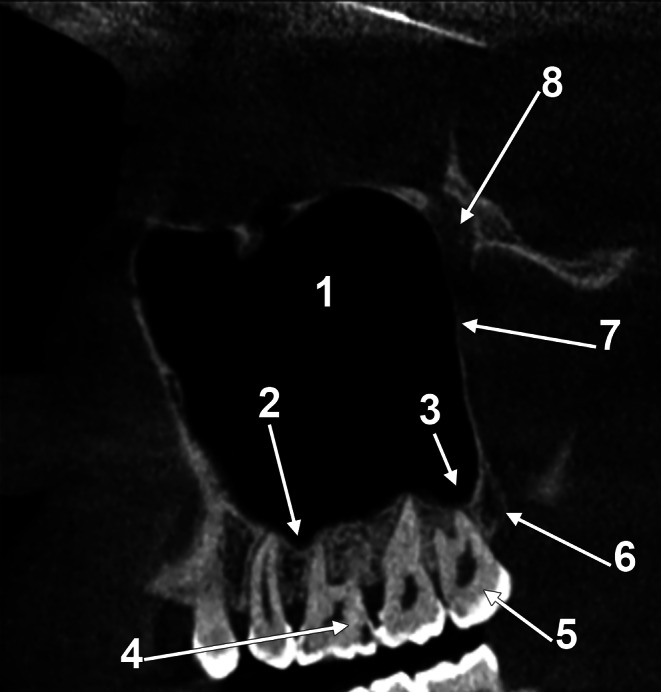
Orthosagittal CBCT slice through the alveolar process of the maxilla. A posterior sinus floor recess enters the alveolar tuberosity. (1) maxillary sinus; (2) mesial basin of the sinus floor; (3) distal basin of the sinus floor; (4) first upper molar; (5) third upper molar; (6) alveolar tuberosity; (7) maxillary tuberosity; (8) pterygopalatine fossa

Confusion is augmented when the distal alveolar bone is referred to as the maxillary sinus. This is because, in our opinion, authors omit the anatomical classification of the latter. The sinus occupies the body of the maxilla, has a variable volume, and may extend recesses out of the body of the maxilla, such as the palatine, zygomatic, or alveolar recesses [26, 38]. The maxillary molars are seemingly closer to the sinus floor than the premolars [11]. The sinus floor may present anatomically variable septa [12, 34]. These septa divide the sinus floor into a series of pockets– the basins of Underwood [19, 34]. These basins construct an alveolar recess if the latter is divided by septa. The vertical relationships between the maxillary sinus floor and the roots of the molars were classified by Kwak et al. (2004) into five types; in types II-V, the sinus floor is located below root apices in various combinations [15]. Even if an alveolar recess descends into the distal maxillary alveolar bone, the AT located distal to that recess should not be considered part of the posterior wall of the central sinus cavity (Fig. 9). The posterior sinus wall lacks cancellous bone. The AT is distant from the pterygopalatine fossa and its lateral entrance– the pterygomaxillary fissure.

### The misidentified maxillary tuberosity in publications

#### The alveolar tuberosity as an insertion site for implants

The broad definition of the so-called MT holds clinical and surgical significance, as it may serve as an alternative site for sinus augmentation procedures and is commonly used as a recipient site for dental implants [14, 37]. Lopes et al. (2015) describe the MT region as the most distal area of the maxillary alveolar process [17]. This is, in fact, the AT. Indeed, the AT may be used as an implant site and has a variable availability [17], which fits to the alveolar bone and not to the maxillary sinus walls.

Bidra et al. (2011) observed that the literature is puzzling because of the use of terminology associated with implants placed in the pterygoid region [5]. These authors reviewed different terms that are used interchangeably, such as „pterygoid implants”, „pterygomaxillary implants”, and „tuberosity implants” [5]. They documented that the term pterygoid implant has been defined by the Glossary of Oral and Maxillofacial Implants [16] as „implant placement through the maxillary tuberosity and into the pterygoid plate”. In contrast, the MT is defined as „the most distal aspect of the maxillary alveolar process” [5]. The respective glossary [16] ignored the Terminologia Anatomica and misplaced the MT onto the alveolar process of the maxilla.

Fukumoto et al. (2022) studied orthodontic miniscrews inserted “in the palatal side of the maxillary tuberosity” [8]. Anatomically, the MT on the body of the maxillary body could not side the palatal vault, which is only sided by the alveolar processes. These authors [8] studied *de facto* miniscrews inserted on the palatal side of the distal end of the alveolar process of the maxillary bone, i.e., the AT. Yamaura et al. (1998) studied the volume of an osseous pillar, which consists of the pterygoid process, the pyramidal process of the palatine bone, and the AT they termed MT [39].

**Fig. 10:**
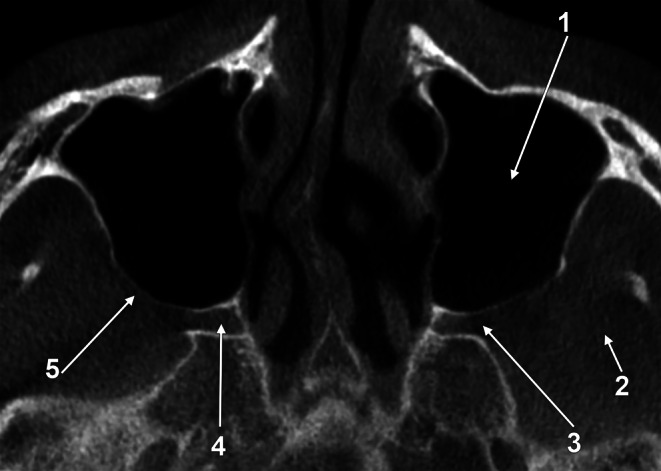
Axial CBCT slice through the maxillary tuberosities. (1) maxillary sinus; (2) infratemporal fossa; (3) pterygomaxillary fissure; (4) pterygopalatine fossa; (5) maxillary tuberosity on the thin posterior antral wall

Graves (1994) described the tuberosity of the maxilla as being composed of type III and type IV cancellous bone [10]. This could not fit for the posterior wall of the maxillary sinus, which is just a thin cortical bone (Fig. 10) but fits for the AT (Fig. 6). This confusion is not trivial. In maxillofacial surgeries, for example, the difference between viewing the tuberosity as part of the alveolar process versus a separate maxillary structure can dictate the success or failure of surgical interventions like pterygoid implants.

When authors assume they studied the so-termed MT covered by the oral mucosa [9], it signifies they studied *de facto* the AT covered by oral mucosa, not the MT on the posterior antral wall. The true AT comprises marrow spaces, adipose tissue, and a low vital bone profile [9]. Indeed, the placement of dental implants in the posterior maxillary area may be problematic, as this is a region where the bone is typically characterised by low-density cancellous bone and thin cortical bone [24]. This applies to the AT.

Lopez et al. (2015) systematically reviewed the placement of implants in the MT [17]. They regarded the MT as „the most distal area in the maxillary alveolar process, posterior to the maxillary sinus” [17]. However, from an anatomical point of view, the distal alveolar bone would never be located topographically posterior to the maxillary sinus. Still, it could accommodate an alveolar recess of the maxillary sinus (Fig. 9). Those authors’ MT appears to be the AT we discuss here. Lopez et al. further described that „bone tissue in the tuberosity region should be less dense than in other areas of the maxilla”, as we demonstrate in Fig. 6, and inserting implants in this region with very spongy bone quality provides predictable osseointegration [17].

#### The alveolar tuberosity as a donor site for grafting procedures

Zucchelli et al. (2020) referred to the MT as a part of the alveolar process, primarily discussing it as a donor site for grafting procedures [40]. Other authors also regarded the donor site for bone grafting as MT, not AT [23, 32, 41].

Our opinion is supported by the study of Khojasteh, Nazeman and Tolstunov (2016), published in the British Journal of Oral and Maxillofacial Surgery [13]. The authors assessed the efficacy of tuberosity-alveolar block bone in augmenting adjacent defects in the maxilla and adequately defined that block as a „posterior maxillary alveolar ridge” [13]. They further established different graft types by referring to the AT and not MT, such as our opinion regarding the distal end of the alveolar process [13]. In those types, the graft from the AT was covered either with titanium mesh, collagen membrane, or periosteum [13]. They finally concluded that bone grafts from the AT may be a good source of bone for augmentation of deficient maxillary edentulous alveolar ridges [13].

**Fig. 11:**
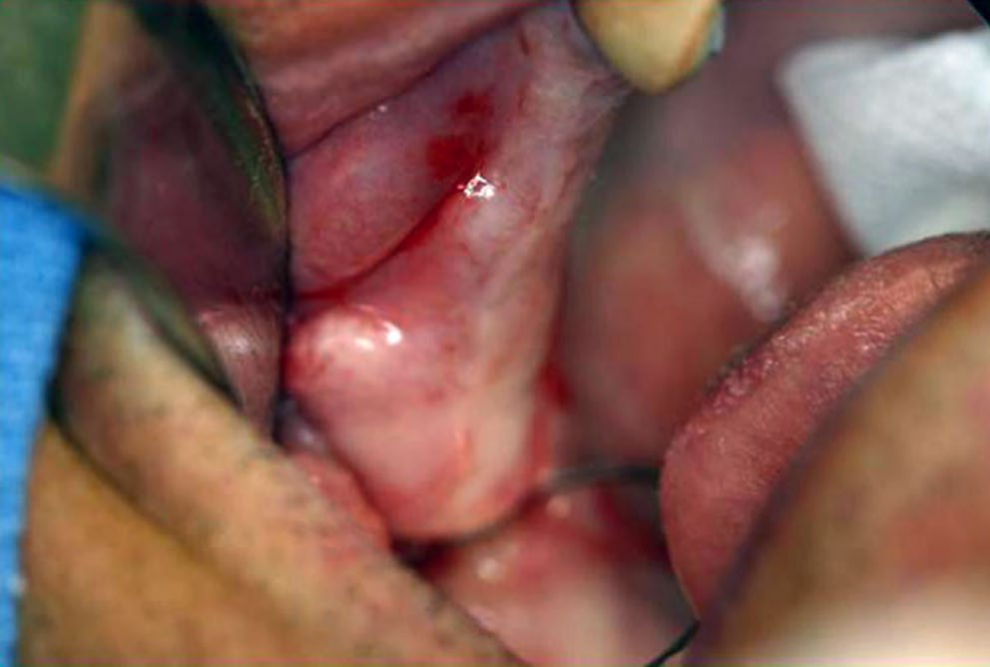
Image reused with the License 5,937,101,196,823/27 December 2024 provided by Elsevier and Copyright Clearance Center, from [32]. The original legend is “FIGURE 3. Preoperative intraoral evaluation showing hypertrophic right maxillary tuberosity”. The distal alveolar bone (alveolar tuberosity) is presented as “maxillary tuberosity”

Zufia and Abella Sans (2022) discussed that Tolstunov was the first to introduce in 2009 the potential of the MT block graft in treating localised maxillary bone defects for implant placement [32, 41]. However, as discussed above, the group Tolstunov made the anatomical update of terms from MT to AT [13]. In that report in 2009, Tolstunov did not locate the bone structure regarded as MT anatomically, but he used the distal end of the maxillary bone (Fig. 11), thus the AT [32]. Anatomically and clinically, the parts of the maxilla and mandible supporting the teeth comprise the alveolar process [23], a specific target for implant insertion or other surgical procedures. Therefore, while the alveolar bone is relevant for surgeons, they may overlook the anatomical MT on the posterior antral wall, which is not used for implant insertion, and erroneously place it onto the alveolar bone.

### Limitations and guidelines for future research

The AT region plays a crucial role in oral surgery. It is increasingly recognised as a valuable source for implants and bone harvesting [9, 13, 17, 24], especially given the preference for using a patient’s bone, or autologous bone, during grafting procedures. Research has shown that this donor site’s bone quality and quantity are essential considerations [4]. Many studies suggest that the bone quantity in the AT is generally quite favourable for treating minor to moderate defects [29].

However, evaluating bone availability in this area comes with its challenges. The bone structure varies significantly, mainly influenced by the presence and status of the maxillary third molar [18], which can cause measurement inconsistencies and make standardisation more difficult. It varies by sex and age [22]. Additionally, the three-dimensional aspect of the AT adds complexity when trying to reproduce reference points from case to case, often leading to approximate measurements instead of precise ones.

Other complicating factors include sample size limitations, the eruption status of the third upper molar, and a notable lack of longitudinal data to track changes over time. Furthermore, current CT/CBCT software algorithms may not fully account for all anatomical variations, which can further complicate volume calculations for irregular morphologies in this region.

Looking ahead, it is vital to maintain strong methodologies using CT/CBCT software for measuring various anatomical landmarks that, in turn, must be accurately termed. There’s also a need for more thorough studies involving more extensive and more diverse samples to truly understand the nuances of the AT. By addressing these limitations, future research can improve our grasp of the morphology of this area and its implications for clinical practice. Consequently, bone availability should be evaluated case-by-case, especially when considering the influence of the maxillary third molar.

## Data Availability

No datasets were generated or analysed during the current study.
